# 4.0 technologies in city logistics: an empirical investigation of contextual factors

**DOI:** 10.1007/s12063-022-00304-5

**Published:** 2022-08-17

**Authors:** Andrea Ferrari, Giulio Mangano, Anna Corinna Cagliano, Alberto De Marco

**Affiliations:** Department of Management and Production Engineering, Politecnico di Torino, Corso Duca Degli Abruzzi 24, Torino, 10129 Italy

**Keywords:** City Logistics, Industry 4.0, Technologies, Regression analysis, Last Mile

## Abstract

Industry 4.0 technologies, originally developed in the manufacturing sector, can be purposefully implemented to improve City Logistics (CL) processes by automatizing some of their operational tasks and enabling real-time exchange of information, with the ultimate goal of providing better interconnection among the actors involved. This work aims to identify the main social and economic contextual drivers for investing in the application of Industry 4.0 technologies to urban logistics. To this end, a dataset based on the primary collection of 105 CL projects exploiting the main 4.0 technologies has been built. After that, a regression model has been completed including potential economic, strategic, and demographic determinants of investments in CL 4.0. According to the obtained outcomes, Gross Domestic Product, Foreign Direct Investments, Research and Development Expenditure, Employment Rate, and Number of Inhabitants are significant contextual factors for the adoption of Industry 4.0 technologies in last mile logistics. The study might support academicians to investigate novel application fields of Industry 4.0 technologies. Also, it can serve as a roadmap for orienting the investments of private organizations and public entities to promote CL innovation and digitalization. Moreover, Industry 4.0 technology providers might find this study interesting to uncover prospective business sectors and markets. Future research efforts will analyse the impacts of internal business factors on CL 4.0 and the satisfaction levels of urban logistics stakeholders.

## Introduction

Goods transportation is a critical process especially in urban areas, since it negatively affects road traffic, air quality as well as infrastructure usage. This adds to the environmental burden imposed by road transportation, whose greenhouse gas emissions accounted more than 70% of the total emissions in the European Union in 2019 (Statista 2021a). In addition, the recent success of B2C e-commerce, which is expected to reach about 6,400 billion $ as worldwide sales in 2024 (Statista 2021b), and the related express delivery and home delivery services, are determining a growing demand for freight transportation services within cities. In this context, the proper management of vehicle fleets, parking areas, together with the monitoring of traffic congestion and in turn of emissions levels, becomes crucial (Kauf 2016).

Therefore, the concept of City Logistics (CL) has been gaining increasing attention (Eshtehadi et al. 2020). It is based on the idea of an integrated logistics system in urban settings associated with coordination and consolidation, and it is aimed at increasing efficiency and reducing the environmental impacts (Rai et al. 2019). Additionally, urban logistics could be referred to as the optimization of logistics and transport activities with the support of performing information systems, taking into consideration pollution, traffic congestion, traffic safety, and energy saving (Taniguchi and Thompson 2014; Taniguchi et al. 2020).

With this regard, the digitalization of logistics may be of the greatest importance. In fact, in the era of digitalization, the use of innovative solutions in urban areas has a positive impact on goods and people flows, resulting in an enhanced life level of quality (Kauf 2019). Additionally, a proper logistics service and technological support have direct influence on economic development of metropolitan networks, thus improving business competitiveness, supporting innovation and encouraging investments (Yang et al. 2019). When it comes to designing and implementing digital and automation supports to logistics activities, the technologies associated with the Industry 4.0 paradigm can be involved. The concept of Industry 4.0 was first introduced in 2011 during the industrial automation fair in Hannover (Germany), aimed to provide products and services with the support of automation and digitalization (Hofmann et al. 2019). In general terms, the objective of 4.0 technologies is the enhancement of machine-human correlation based on an IT-drive transformation (Moosavi et al. 2021). By introducing these technologies, it will be easier to make systems resilient and in a real-time manner (Vaidya et al. 2018). The adoption of 4.0 technologies, which include IoT, Big Data Analytics, Cloud Computing, Artificial Intelligence, Blockchain, Intelligent Transportation Systems, Autonomous Vehicles, Advanced Automation, and Additive Manufacturing (Paiva et al. 2021), might play a crucial role in optimizing the logistics service, thus implementing more integrated and dynamic solutions.

In such a context, the notion of Logistics 4.0 emerges as networking the whole supply chain through information technology, wherein sensors and automated robots are used in operations (Jahn et al. 2018). It involves system-based planning and control of the physical movement of goods and the associated information flow from source to destination, by using front end and base technologies to enhance the customers’ requirements (Frank et al. 2019). All these definitions can be summarized by the definition provided by Winkelhaus and Grosse (2020): “Logistics 4.0 is the logistical system that enables the sustainable satisfaction of individualized customer demands without an increase in costs and supports this development in industry and trade using digital technologies”.

Based on such a notion, City Logistics 4.0 (CL 4.0) is the application of Logistics 4.0 to urban transportation (Taniguchi et al. 2020). Its importance has increased due to the rise of the e-commerce over the past 20 years, and especially during the lockdowns related to the COVID-19 pandemic (Correia et al. 2021). Thus, CL systems are facing relevant criticalities, and the application of innovative technologies might be beneficial for improving the effectiveness of operations and at the same time for enabling sustainability (Torbacki and Kijewska 2019). Additionally, again in the light of the recent events, increased resilience capability and flexibility are required to CL, together with an even more customized attention to the people that either benefit from its services or are impacted in some way by them. So, digital technologies, under the umbrella of not only the Industry 4.0 notion but also of the newer Industry 5.0 one, find a lot of room for implementation. In fact, the main aim of Industry 5.0 is applying technology with the purpose to achieve human-centred, sustainable and resilient processes (Xu et al. 2021).

Few academic contributions study the use of Industry 4.0 technologies in last-mile logistics and analyse the impact of the socio-economic configuration of urban areas on the willingness to invest in innovative CL projects. In fact, most of the literature is focused on the assessment of CL projects (Cleophas et al. 2019), evaluating their operational and economic feasibility (Björklund and Johansson 2018). Thus, especially company operational and economic elements are taken into account. For example, cost aspects related to the human resources, facility, maintenance, and rent are considered. Then, the consequent impacts for the citizenship (Tadić et al. 2018) are discussed, keeping in mind that the different stakeholders interacting in a CL system have different goals to be achieved. In fact, individual objectives are not always aligned, and what is perceived as positive by one group, might cause several negative effects to other ones. Finally, CL initiatives are evaluated according to different time spans (short, medium, long run), in order to point out related suitable approaches and solutions (Cleophas et al. 2019). Another stream of literature research deals with the application of 4.0 solutions to CL and it is mainly focused on the viability of innovative freight transport projects from the economics and operational points of view (Korczak and Kijewska 2019). The main aspects addressed refer to the adoption of new logistics and distribution approaches (e.g. off-peak deliveries; collaboration schemes), use of innovative technologies for an accurate exchange of information, promotion of greener practices, such as low impact vehicles and monitoring traffic systems.

Thus, there is a lack of contributions assessing the role of contextual variables related to the urban, political, and operations environment wherein an innovative CL initiative is developed, intended as a project carried out in order to search solutions for the problems related to urban freight transport activities (Kijewska 2019). This aspect is quite important since it allows to evaluate the economic and socio-political contexts wherein CL projects are implemented (Cagliano et al. 2021). In fact, the urban structure might play a role in affecting the propensity to invest in CL systems. Moreover, the policy makers might be more able to deal with new logistics projects at an urban level. Consequently, it would be possible tracing their actual influence on the willingness to develop CL innovative programs. This aspect is acquiring a relevant importance considering that in several areas of the world (for instance Europe), public authorities are significantly supporting innovative projects that are expected to improve the quality of life of citizens from an environmental point of view.

To this end, in order to bridge the outlined research gap, the objective of the present work is to deepen the understanding of the application of Industry 4.0 to CL and, specifically, to determine the potential contextual factors that might drive the willingness to invest in 4.0 technologies in urban logistics settings. To this end, the main 4.0 initiatives so far applied to CL are here considered. However, it has to be stated that the diffusion is CL 4.0 is nowadays limited, since the associated technologies are still largely applied to the different manufacturing industries (Raut et al. 2020). Therefore, in this work, the monetary investments related to a set of projects, aimed to implement 4.0 technologies in the context of urban logistics and carried out in the last decade, have been considered in order to analyse and identify which might be the external drivers that can facilitate the development of this kind of solutions.

The remainder of this paper is structured as follow. Literature review illustrates the research background on CL concept related to Industry 4.0 and the innovative technologies applied to distribution processes in urban areas. Then the methodology adopted in this study is introduced by describing the construction of the dataset used and the empirical analysis conducted. After that, the discussion of the results obtained is presented. Finally, conclusions complete the article with possible academic and practical implications, the limitations of the study, and future research streams.

## Literature Review

The growth of the number of commercial vehicles in municipalities, mainly due to the recent increase in urbanization and the broader diffusion of e-commerce, is not always associated with the development of proper infrastructures and transport planning strategies (Kummer et al. 2021). For this reason, there is a raising need to evaluate improved and innovative solutions able to reduce emissions and optimize last mile distribution routings (Russo and Comi 2018). Additionally, in supply chains, the last mile still represents a significant portion of the total delivery costs (Ranieri et al. 2018). Thus, it is fundamental for companies to maximize the efficiency of urban logistics operations. Likewise, governments and local authorities seek to optimize urban freight transportation since it contributes to deteriorate the environment and citizenship well-being conditions (Leyerer et al. 2019). In such a context, the CL processes could benefit by digital systems that enable almost real-time interaction between customers and suppliers for the proper handling of orders (Cagliano et al. 2017b; Mangano et al. 2019). In the last decade, the digital evolution provided by the Industry 4.0 technologies is going to become a lever for value creation (Frederico 2021), and their implementation is a source of innovation in supply chain environments (Munksgaard et al. 2014). At a general urban level, Industry 4.0 technologies are factors for increasing interconnectivity among buildings, grid and mobility, and to support policy makers and logistics providers in satisfying customers’ expectations (Tang and Veelenturf 2019). Also, modelling CL by exploiting them has been proved to be more and more promising (Taniguchi et al. 2020).

This literature review aims to provide an overview of the most relevant 4.0 technologies that have been applied in urban logistics processes. In order to determine the entire list of innovative 4.0 technologies employed in the CL context, a research was conducted by using the Scopus database, as it is broadly considered as a standard in the academia (Bag and Pretorius 2020). The search was limited to four keywords, namely *city logistics*, *urban logistics*, *last mile logistics* and *last mile*. Only generic keywords were selected in order to include as many publications as possible about urban logistics and to provide a comprehensive overview regarding the topic of CL 4.0. Moreover, only papers published from 2011 on were included as Industry 4.0 notion was established in that year. An initial number of 856 papers was obtained.

These papers resulting from the search queries were selected by reading the title, the abstract and, if necessary, the entire article. In this preliminary exploratory phase of the literature review, all articles describing the introduction or analysing the application of an Industry 4.0 technology in the field of urban logistics were selected. As a consequence, articles having as main topic logistics network design, process and routing optimisation, sustainability, policy introduction, impact assessment and logistics performance definition were then excluded. This process allowed to accurately identify the list of enabling Industry 4.0 technologies that find application in the urban logistics sector. Finally, for each technology found, its main applications were analyzed and here presented.

The digital transformation that logistics networks are facing is offering new opportunities to integrate and increase the efficiency of the IT tools traditionally supporting CL, such as Intelligent Transportation Systems (ITS) or on-board and road-side units detecting vehicle and traffic performance. New technologies like Internet of Things (IoT) and Big Data are able to improve good traceability and communication among vehicles, as well as to quickly process a large amount of information. Thus, it is possible to predict the future demand for CL activities more accurately in order to optimize the associated service level.

Additionally, problems related to the complexity of current urban logistics and its effects on traffic congestion and pollution can be more easily addressed (Florence and Shyamala Kumari 2019; Yavas and Ozkan-Ozen 2020). A smarter management of goods through routing efficiency, vehicle loading strategies and more effective customers communication (Torbacki and Kijewska 2019) can also contribute to better manage logistics risks (Kamp and Gibaja 2021).

Therefore, through Industry 4.0 innovative technologies, logistics is becoming a lever for competitive advantage and it is no more merely considered as an unavoidable cost burden (Tang and Veelenturf 2019). Among the pool of Industry 4.0 technologies, the most relevant ones adopted in urban contexts are listed and defined in Table 1.

**Table 1:** Definition of Industry 4.0 technologies adopted in CL context

Industry 4.0 Technology	Definition	References
Information and Communication Technology (ICT), Smartphone applications, and Intelligent Transportation Systems (ITS)	Combination of sophisticated technology and innovations in information systems, communication, sensors, controllers, and advanced mathematical methods for accessing, managing, and transmitting information in a digital form within the context of transportation infrastructure. Use of dedicated smartphone applications to place orders and manage the delivery of goods.	Lin et al. 2017; Ahad et al. 2020
Internet of Things (IoT)	Deployment of sensors and devices interconnected through telecommunication networks and Internet-based communication aimed at incrementing product and processes value.	Golpîra et al. 2021; Peraković et al. 2019; Witkowski 2017
Big Data	A very high volume of data characterized by high speed and large variety managed using innovative analytics techniques in order to support decision-making.	Choi et al. 2018; Zhu 2018
Cloud Computing (CC)	Online service enabling the possibility to perform quick and lighter computations instead of setting up a physical infrastructure. It allows a set of computing resources, namely networks, servers, storage, applications, and services, to access to an on-demand, ubiquitous, and useful network. It results in a cost effective and faster solution in terms of operating platforms, software, infrastructures.	Ahad et al. 2020; Bhardwaj et al. 2010; Golpîra et al. 2021
Artificial Intelligence (AI)	Solution deploying sophisticated algorithms and techniques aimed at processing and analysing the high quantities of data generated from machine-to-machine communication in order to derive sense and valuable information.	Ahad et al. 2020; Allam and Dhunny 2019; Bertolini et al. 2021; Paiva et al. 2021
Drones and Autonomous Vehicles	Vehicles able to autonomously and automatically perform precise operations thanks to the implementation of sensing systems. They can move safely with minimum or absent human input.	Baum et al. 2019; Müller et al. 2019; Yu et al. 2021
Blockchain	A digital platform allowing a group of users to securely store and distribute information by creating time-stamped, tamper-proof, and perpetual records. It consists of decentralized ledgers containing transactions as data blocks, linked among each other through a cryptographic pointer. It is characterized by distributed consensus, secure, traceable, verified, and transparent information.	Bekrar et al. 2021; Kouhizadeh et al. 2021; Mukherjee et al. 2021.
Additive Manufacturing (AM)	A set of manufacturing technologies and processes for fabricating a wide range of geometries and structures from digital three-dimensional models by printing successive layers of materials that are formed on top of each other. It allows increased degree of freedom in design, mass customisation, waste minimisation, productions of complex structures, and faster prototyping.	Alogla et al. 2021; Ngo et al. 2018; Zunder et al. 2014

Information and Communication Technology (ICT), ITS, IoT, Big Data, Cloud Computing, and Artificial Intelligence (AI) are significant for establishing the CL paradigm, allowing an integrated management platform of urban freight transport systems (Taniguchi et al. 2020).

Although the notion of ICT was introduced well before the Industry 4.0 one, its recent developments can be considered as the main trigger of the Fourth Industrial Revolution, which relies on the combination of various ICT tools with the ultimate goals of connecting assets and facilities, explaining data, and digitizing business operations (Peraković et al. 2019). Therefore, ICT can be included among the Industry 4.0 technologies. The combination of ICT with IoT in supply chains provides a widespread information infrastructure, thus obtaining performance improvements and energy consumption reductions (Garrido-Hidalgo et al. 2019). Another prominent implementation of ICT tools in CL is given by mobile applications to manage good ordering and receiving. In such a context, a dramatic increase of the use of smartphone applications for the urban food delivery can be observed and the recent outbreak of COVID-19 induced a further surge in using these apps (Ortiz-Prado et al. 2021; Smith et al. 2021). Basically, restaurants determine the on-line food price and platforms add a logistics fee for the final delivery process (Niu et al. 2021).

Furthermore, a better management of urban systems and last-mile operations is possible through AI and machine learning technologies, which provide a deeper understanding of how cities evolve, adapt, and respond to various conditions (Allam and Dhunny 2019). By applying AI, logistics networks will adopt more proactive, predictive, automated, and personalized strategies able to increase the overall performance of the distribution system (Gesing et al. 2018).

Urban Logistics 4.0 also comprises solutions like the use of Drones and Autonomous Vehicles (Bechtsis et al. 2018), supports for Blockchain development (Abeyratne and Monfared 2016), and Additive Manufacturing applications (Heutger and Kückelhaus 2016).

The theme of Autonomous Vehicles rises particular interest in the CL context, since they can increase safety in freight activities. Additionally, they might influence urban goods movement in terms of sustainability, savings, quickness, and customers satisfaction (Baum et al. 2019).

Also airborne Drones represent a promising technology for parcel delivery but their successful implementation depends on their actual economic benefits, uncertainties related to their technical features, and socio-environmental impacts (Kellermann et al. 2020).

Additionally, CL is changing thanks to the adoption of Blockchain technology, because it enables cost reduction, quality enhancement, and improved efficiency (Mukherjee et al. 2021). To be more precise, this technology has great potential to sustain smart and traceable packaging and support tracing of materials in reverse logistics operations (Dutta et al. 2020). In the context of micro-hubs and last-mile deliveries, Blockchain represents a potential solution to address the lack of trust and data issues (Hribernik et al. 2020).

Finally, Additive Manufacturing has a significant impact on urban environment logistics, especially in the spare part industry, by manufacturing individualized products and allowing on demand production and new customer-centric solutions.

Table 2 summarize the main CL applications of the Industry 4.0 technologies at issue.

**Table 2:** Industry 4.0 technologies applications in CL context

Industry 4.0 Technology	Applications	References
Information and Communication Technology (ICT) and Intelligent Transportation Systems (ITS)	Traffic monitoring to increase road safety and efficiency, especially in dense urban road networks	Boukerche et al. 2020
Internet of Things and Big Data	Applying IoT to obtain Big Data of logistics resources and determine the best route consistent with the delivery requirements. Monitoring traffic congestion, parking spaces, state of roads, and traffic problems via IoT technology. Enabling dynamic optimization of reverse logistics, providing real-time data, exploitable to diminish total logistics costs, energy consumption, and total logistics distance, as well as to improve the configuration of logistics resources and to achieve a more sustainable service.	Liu et al. 2018; Witkowski 2017; Zhu 2018
Cloud Computing	It improves CL processes by enhancing information sharing internally and externally throughout a supply chain network. It accelerates information transfers among the actors of logistics networks, thus helping solve the problem of increasing transport costs and in-time delivery requirements. It ultimately assists in designing an adaptive CL infrastructure in relation to changing transport demand, in order to enhance the efficiency level.	Cao et al. 2017; Niharika and Ritu 2015; Nowicka 2014
Artificial Intelligence (AI)	The application of AI to transportation logistics concerns infrastructure and traffic organization, automated and autonomous driving, predictive maintenance, and route and transport organization. It provides autonomous decisions and predictions about road conditions, traffics, and streetlight.	Loske and Klumpp 2021; Paiva et al. 2021
Drones and Autonomous Vehicles	Groups of drones and autonomous vehicles integrated with trucks to execute last mile deliveries. They are carried by a truck and moved to a dispatch point from where they perform deliveries to single customers. Trucks replenish drones and autonomous vehicles for the next deliveries.	Chung 2021
Smartphone applications and freight services	Placing orders, planning and choosing transportation routes, acquiring real time information on the availability of urban facilities (e.g. lay-bay areas) and traffic conditions. Smartphone applications to order food products from restaurants or retail shops that are then delivered using a fleet of bikes or electric scooters.	Cagliano et al. 2015; Iwan et al. 2014
Additive Manufacturing (AM)	Application to last-mile spare parts logistics, supporting strategies such as low emission areas, adoption of urban consolidation centres, and sustainable vehicle fleet management.	Heutger and Kückelhaus 2016; Zunder et al. 2014

The overview of the literature highlights that 4.0 technologies are playing a crucial role in CL systems in term of efficiency and effectiveness of the related processes. However, most of the available literature on logistics 4.0 does not specifically address CL (Efthymiou and Ponis 2021; Winkelhaus and Grosse 2020). Additionally, although the first existing contributions on the implementation of Industry 4.0 technologies to facilitate urban logistics processes, the current CL literature is still mainly focused on the feasibility of urban transportation projects from both an operational and a financial point of view, without any emphasis on the main contextual drivers of the application of innovative 4.0 technologies. In particular, empirical studies on this topic are still scarce. As a matter of fact, the literature debate on the enabling factors for 4.0 technologies is nowadays largely limited to the manufacturing sector and the associated industrial facilities (Longo et al. 2019; Raut et al. 2020).

So, there is a need for academic works concentrating on the determinants of the implementation of digital technologies in CL based on the key urban, political, and operational features characterizing each application. In fact, probably even more than in other contexts, the viability of CL projects is highly dependent on the conditions of the city and the geographical area at issue, such as for instance the associated economic prosperity, the willingness to invest in innovative solutions, and the social conditions of population (Neghabadi et al. 2019).

For this reason, in the present research a widely accepted empirical approach has been applied with the aim of revealing and understanding the significant dependence of CL implementations on the above-mentioned external conditions.

## Methodology

### Construction of the database

The objective of this research is to identify the main potential contextual factors guiding the investments carried out to develop innovative logistics solutions in cities context. Therefore, a dataset including the principal applications of innovative solutions to urban logistic was built by selecting one by one CL projects exploiting 4.0 technologies. The primary sources used for building the dataset were different. For each of them, a research was conducted searching for terms like *city logistics*, *urban logistics* and the name of the different *4.0 technologies* previously identified in the literature review and listed in Table 1. First, suitable scientific papers analysing CL 4.0 implementations were identified using the Scopus database. Second, the research was extended by analysing any reports and documents in the references of these articles. Finally, the project research was further expanded looking for CL solutions exploiting 4.0 technologies. In this phase, data were gathered by using public and business reports and websites like Crunchbase, Skift Table, Pagan Research, Tracxn, Start-up Nation Finder, TechCrunch, and Indiegogo. For each observation found, a careful analysis was conducted. The projects in which the monetary investment carried out was missing were excluded. Then, also project regarding CL but not involving the implementation of innovative 4.0 technologies were ignored.

For each project included, the name of the application, a brief description, the country and year of implementation, the economic value of the investment and the main 4.0 technology used were traced.

Additionally, since the objective of the analysis is to establish the relationship between the implementation of 4.0 logistics solutions in urban areas and contextual factors characterizing the geographical area of development, a set of variables were selected in order to frame the socio-economic structure of the areas of implementation of the projects. These were GDP, GDP growth, Foreign Direct Investments, R&D Expenditure, Employment rate, People with a Bachelor’s Degree, Population Density, Number of Inhabitants, and Internal Private Credit. They were identified by literature investigation aimed to demonstrate their relevance for fostering the willingness to invest.

Indeed, GDP, used as a monetary measure, shows the market value of all the final goods and services produced in a certain period of time. Since it reflects the economic development of a country, it can be considered as a lever for fostering innovative technologies (Fan 2011). In addition, the GDP growth was taken into account as a proxy to assess the speed of economic development (Naveed et al. 2018). Similarly, the GDP per capita, computed as the ratio between the GDP and the total number of residents, was included as a factor able to support the diffusion of innovation (Akhavan et al. 2020). Thus, it is expected that the higher this variable, the higher the availability of financial resources and in turn the willingness to invest in innovation.

The Foreign Direct Investments (FDI) are structured long run investments carried out between a foreign organization and a hosting country; these typically bring new technologies and know-how (Osei and Kim 2020). Therefore, it can be assumed that broader FDI brings increased investment in urban logistics projects.

Investments in Research and Development (R&D) are aimed at creating knowledge diffusion and innovation (Jang 2019). From this perspective, significant R&D budgets are likely to lead to more financial engagement in new urban logistics initiatives.

The Employment Rate was also considered in the proposed study because a large number of people with a job might be a strong trigger for innovation (Aubert-Tarby et al. 2018).

The Population that has gained a Bachelor’s Degree was another crucial variable. As a matter of fact, it is more and more considered an important pillar of technological innovation and upgrade (Wu and Liu 2020).

In urban contexts, another variable that might support innovation is the Population Density, expressed as the ratio between the number of inhabitants and the squared kilometres of the area considered. In fact, higher densities might be related to higher inclination to undertake smart innovative programs aimed to deal with the current urban open issues such as traffic congestions and environmental pollution control (Caragliu and De Bo 2019). The Urban Population, considered as the percentage of people living in cities over the total country population, was also included because for countries with wide urban areas there are typically more business opportunities for promoters and project investors in new technological initiatives (Lan et al. 2020).

Finally, the Private Credit was taken into account because, as it is associated with the amount of financial resources provided by private investors, it represents a potential lever in supporting the development of innovative investments (De Marco and Mangano 2013).

The present set of variables can be considered exhaustive for capturing the big picture of a city context wherein promoters might find some potential business opportunities. In fact, the business aspect is included in most of the considered variables such as the GDP, the Population Density, and the Employment Rate. In particular, the identified variables include the different aspects related to the urban context wherein a project might be implemented. Specifically, three main dimensions are considered, namely Economic, Social, and Urban Structure variables (Table 3).

**Table 3:** Classification of contextual variables

Category	Variables
Economic	Gross Domestic Product (GDP) GDP growth GDP per capita Foreign Direct Investments R&D Expenditure Internal Private Credit
Social	Employment rate Population with a Bachelor’s Degree
Urban structure	Population Density Number of Inhabitants

Based on the city or country were the project was implemented, the data related to these socio-economic variables considered were collected by exploiting public databases, namely, Census, Statista, and World Bank. Some of these variables were associated with the urban environment wherein the project was carried out. Other ones were collected at the national level and were referred to the country. This information represented a proxy for capturing the big picture of the context wherein the innovative initiative was promoted. Thus, the dataset has been developed project by project.

### The empirical analysis

In order to study a possible relation between the socio-economic configuration of urban areas and the implementation of Industry 4.0 projects in CL sector, the methodology applied in this study consisted of a linear regression analysis. This approach was selected as the most suitable one since it is broadly used with continuous variables for capturing potential dependencies between a dependent variable and other independent predictors (Hedeker and Gibbons 1994). In addition, it proves its suitability for estimating how different variables can affect a dependent variable (Cagliano et al. 2017a), as it tests if the independent variables taken into account are statistically significant factors and whether they have a positive or negative impact on the response variable. A positive influence indicates that an increase (or decrease) in the independent variable determines an increase (or decrease) in the dependent variable. On the contrary, a negative effect determines opposite direction between independent and response variable variations. The level of significance for each independent variable is related to the p-value, which ranges from 0 to 1 and is obtained from the regression model. If the p-value is lower than a critical threshold, that is usually equal to 5%, the null hypothesis has to be rejected and thus the independent variable at issue can be considered as significant. In other words, the notion of significance is not related to the strength of the relationship but to its reliability. Based on the dataset obtained, the identified response variable of the proposed regression analysis was the investment required, in terms of Capital Expenditure, to implement a CL 4.0 technology project. The investment variable was taken into account because it measures the financial engagement required to initiate a project (Katsela and Pålsson 2021) and it can be considered for assessing the attention of both public agencies and private promoters on CL systems (Montwiłł et al. 2021). Optimization of logistics activities, changes in the urban transport systems, and the implementation of mobility policies call for financial contributions depending on the nature of the development (Zysiñska 2020). Additionally, the accomplishment of CL initiatives relies on the willingness of private organization or local authorities to invest in the value offered by the project (Quak et al. 2014). These issues demonstrate both the relevance of the economic investment and the role played by the urban context wherein the initiative is carried out. On the contrary, the independent variables in the present study were the socio-economics factors that are likely to influence the investment strategy, namely the contextual variables listed in Table 3.

Before carrying out the regression analysis, the investigation of the multicollinearity among the predictors was conducted. This was performed through the computation of the Variance Inflation Factor (VIF). It evaluates the relationship between an independent variable and all the other ones and it is obtained as 1/(1-R^2^), where R^2^ is the coefficient of determination of one variable on all the others and it expresses the portion of variance related to the independent variable at issue that can be referred with the other independent ones considered in the study. Variable showing a VIF greater than 5 are one by one discarded because the regression coefficient is poorly estimated (De Marco and Mangano 2017). Then, the new VIF values are again computed. After that, the linear regression model was conducted and the statistically significant independent variables were identified.

In addition, in order to directly consider in the analysis both the continent and the technology adopted for each project, an Analysis of Variance (ANOVA) was carried out. This empirical approach was based on a hypothesis test in which for the different categorical groups at issue, the median is considered. The null hypothesis is that all the values of the mean are equal. On the contrary, if the resulted p-value of the test is lower than the critical threshold of 5%, the null hypothesis has to be rejected. Thus, this means that at least one mean is significantly different compared with the other ones (Cagliano et al. 2021).

**Fig. 1:**
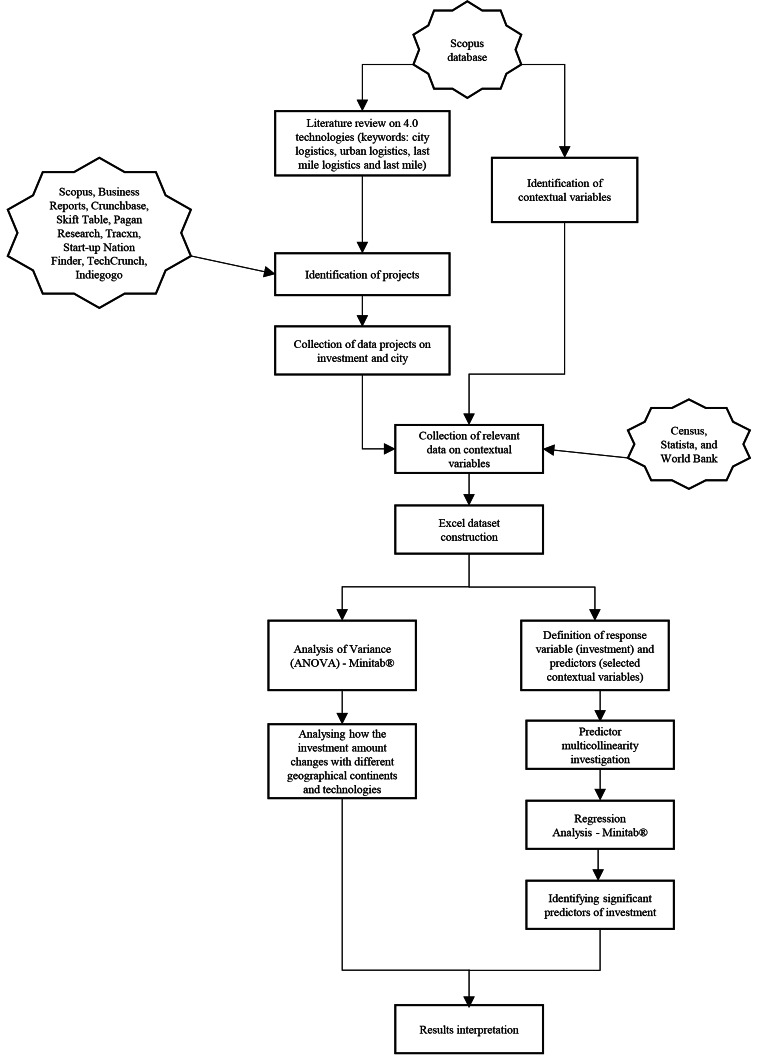
Methodological Steps

Both the methodological steps regarding the construction of the database and the empirical analysis conducted are summarized in Figure 1.

## Analysis of results

### Description of the dataset

The dataset has 105 records. The projects included in the dataset correspond to solutions developed in the last decade, and more than half are less than 5 years old. As already mentioned, the data have been collected via primary open sources and reports showing the implementation of innovative CL projects with the information related to the investment required for developing the project.

**Fig. 2:**
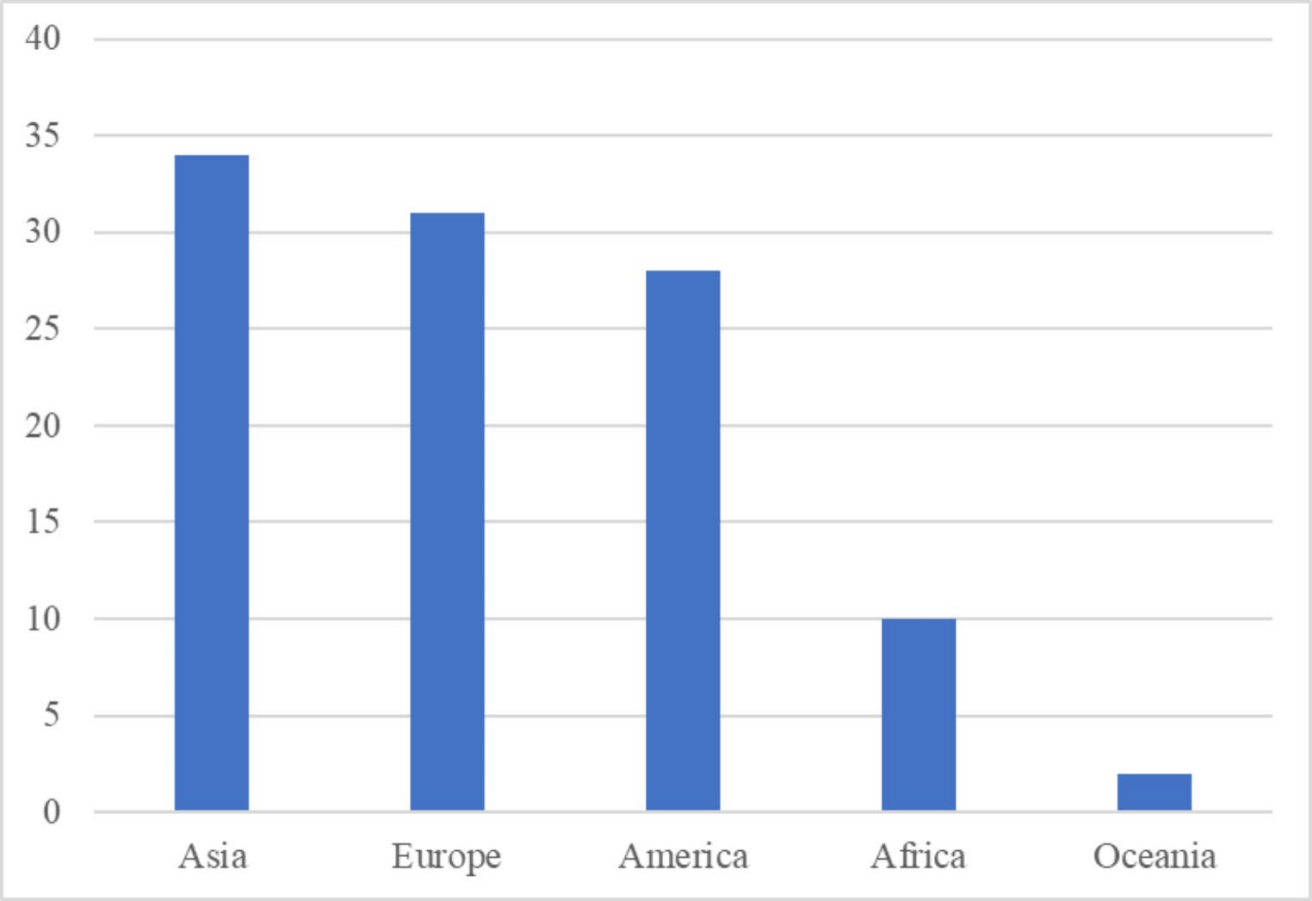
Geographic distribution of 4.0 CL projects

Regarding the geographical distribution of 4.0 CL innovations, it can be observed that most of the projects operates in Asia, followed by Europe and America (Figure 2). It is worth underling that the number of implementations in Africa reflects the growing demand for logistics operations in emerging countries and the tendency of foreign promoters in investing in these promising areas of the world (Werikhe and Jin 2016). On the other hand, Oceania is the continent that shows the least incidence of urban logistics projects.

**Fig. 3:**
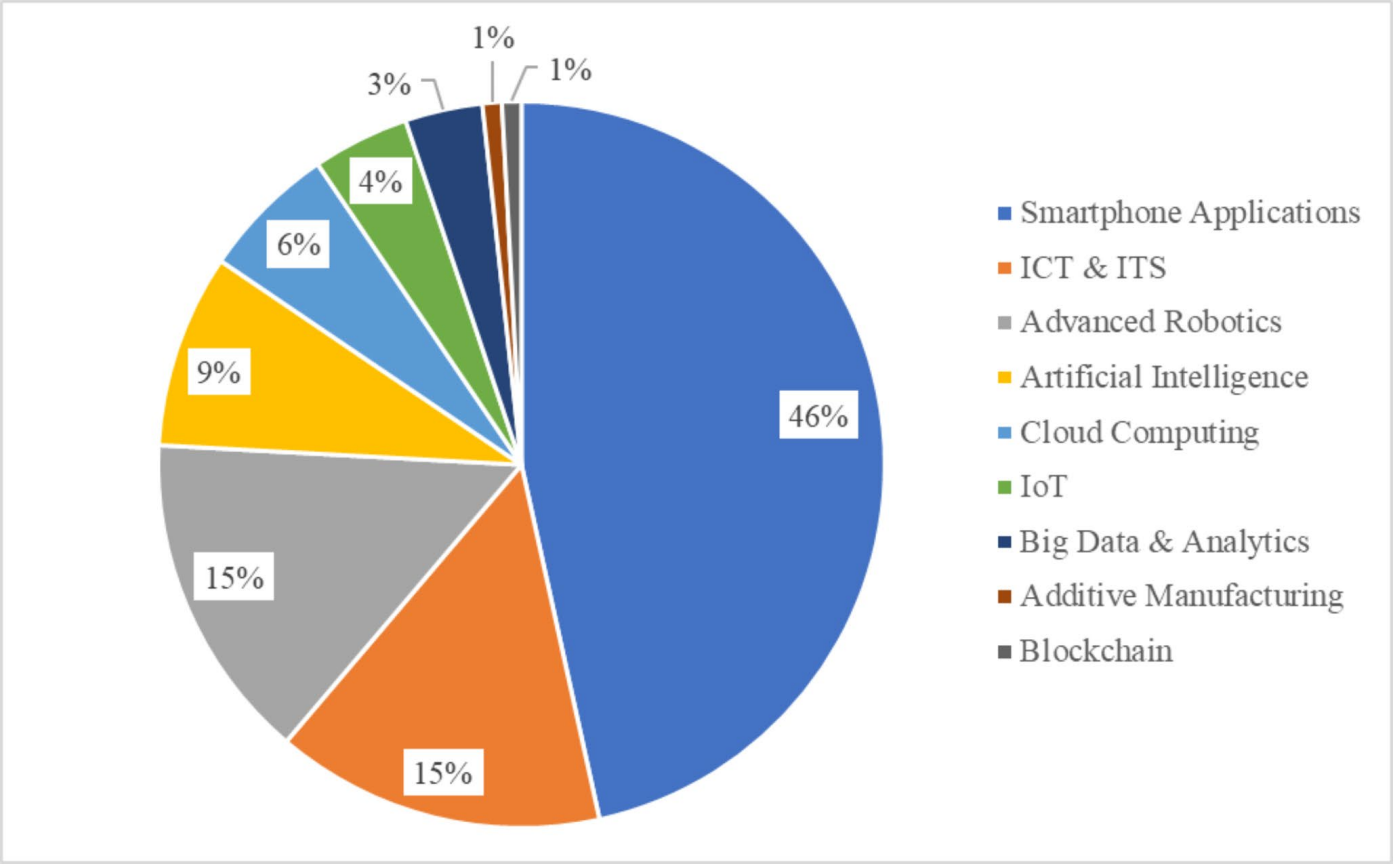
CL 4.0 Technologies

Concerning the innovative technologies implemented in urban context, some preliminary analyses are carried out in order to facilitate the interpretation of the outcomes of the regression analysis. The dataset shows a large diffusion of Smartphone Applications, mostly related to food delivery projects. This result mirrors the increasing trend in the use of mobile food apps due to higher busy and dynamic routines of consumers, which more often prefer to order food online rather than cooking at home (Vinaik et al. 2019). The second most used solution is ICT, followed by Advanced Robotics, Artificial Intelligence, Cloud Computing, IoT and Big Data & Analytics. The 4.0 technologies that most struggle to take root in urban environments are Additive Manufacturing and Blockchain (Figure 3).

Regarding the average investment carried out to implement related projects (Table 4), the first place achieved by ICT & ITS, in conjunction with its positioning in terms of diffusion, presumably relies on the fact that ICT consists of different transversal solutions combined together with the aim of digitizing business operations (Peraković et al. 2019). Furthermore, ITS is a quite established technology in Industry 4.0 and CL context. In addition, it is interesting to note how, Smartphone Application technology only positions at the fifth place, despite the broadest diffusion of such implementations in urban logistics context. On the contrary, even though Additive Manufacturing solutions seem to have difficulties in being used in CL sector, they show high level of investments probably due to the newly and costly technology. The same trend can be observed for Big Data & Analytics and IoT implementations. Finally, Artificial Intelligence, Cloud Computing, and Blockchain show the lowest levels of investment of the list.

**Table 4:** Average investment for each technology

Technology	Average Investment (M $)
ICT & ITS	99.15
Additive Manufacturing	67.30
Big Data & Analytics	66.10
IoT	65.50
Smartphone user application	44.22
Advanced Robotics	34.39
Artificial Intelligence	9.37
Cloud computing	7.26
Blockchain	5.40

### Empirical analysis

Before performing the linear regression analysis aimed at understanding a possible relation between the willingness to invest in CL 4.0 projects and the contextual drivers of the area of implementations of the projects, it is fundamental determining if there is multicollinearity among independent variables included in the study. Table 5 shows that multicollinearity exists in the model and in particular Foreign Private Investment has the highest VIF. Thus, this predictor is removed to avoid multicollinearity; as shown in the right side of the table, after such an operation each VIF is lower than the critical threshold.

**Table 5:** Multicollinearity in the model

Term	VIF	Final VIF
GDP	5.62	2.85
GDP Growth	1.75	1.72
GDP per Capita	4.7	4.57
**Foreign Direct Investment**	**10.79**	**-**
R&D Expenditure	3.02	3.02
Employment Rate	1.15	1.15
Population with Bachelor’s Degree	3.88	3.83
Population Density	1.59	1.56
Urban Population	4.04	3.90
Internal Private Credit	4.16	3.94

**Fig. 4:**
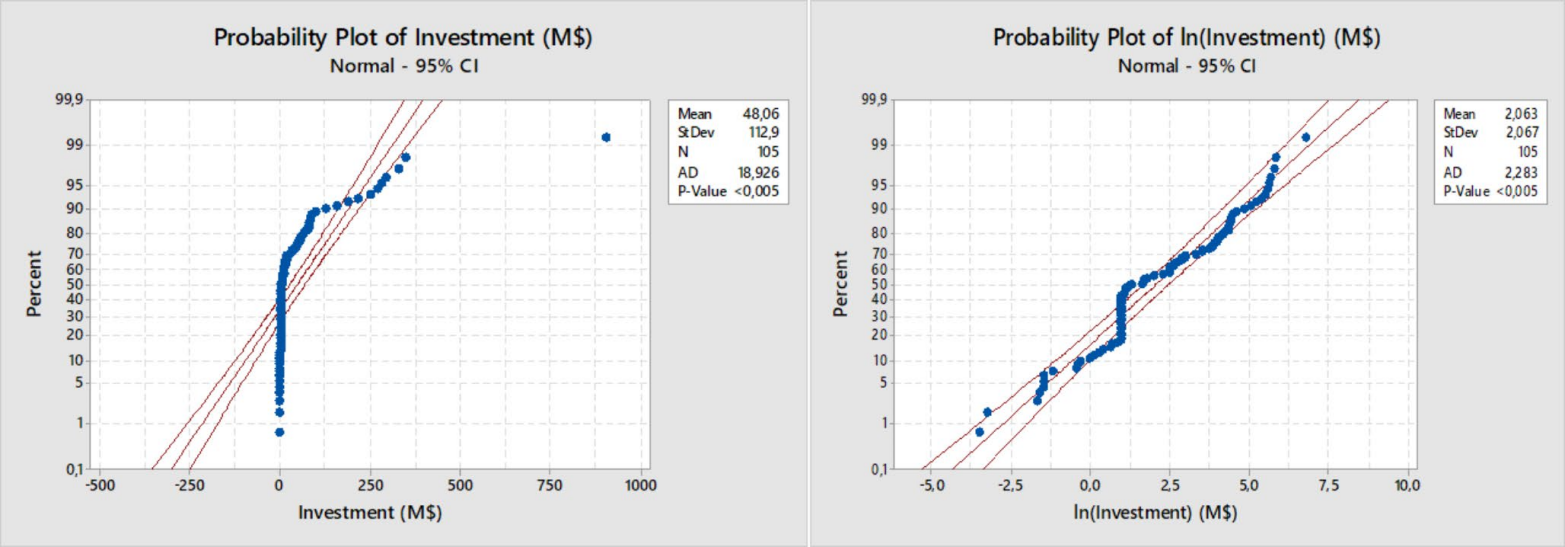
Normal Probability Plot before and after the Logarithm Transformation

After the study of the multicollinearity, the regression analysis is completed. This statistical model is not applicable because the Normal Probability Plot does not show the normality of records (Figure 4). Thus, a logarithm transformation on the response variable is carried out. Now the probability plot highlights the normal distribution of data (Figure 4).

Once the normal distribution of data is obtained, it is possible to perform the linear regression analysis. Its outcomes are presented in Table 6. For each independent factor included in the statistical model, the coefficient together with its standard error, the T-value, and the P-value are shown.

**Table 6:** Results of the Regression Analysis

Term	Coefficient	SE Coef	T- Value	P-Value
Constant	7.32	1.47	4.97	0
GDP	0.000001	0.000001	2.03	**0.045**
GDP Growth	0.0044	0.08	0.05	0.956
GDP per Capita	0.000041	0.0000015	2.65	**0.010**
R&D Expenditure	-0.643	0.302	-2.13	**0.036**
Employment Rate	-0.0677	0.0188	-3.61	**0.00001**
Population with Bachelor’s Degree	0.0023	0.0325	0.07	0.975
Population Density	-0.000174	0.000127	-1.36	0.176
Urban Population	-0.0359	0.165	-2.17	**0.032**
Private Credit	0.00587	0.00562	1.04	0.3

The level of significance of the independent variables depends on the p-value. In particular, the variables showing a p-value lower than 5% can be considered as significant. Consequently, in the model proposed, the P-value underlines that GDP, GDP per capita, R&D Expenditure, Employment Rate and Urban Population are significant drivers to invest in 4.0 technologies applied to CL.

**Fig. 5:**
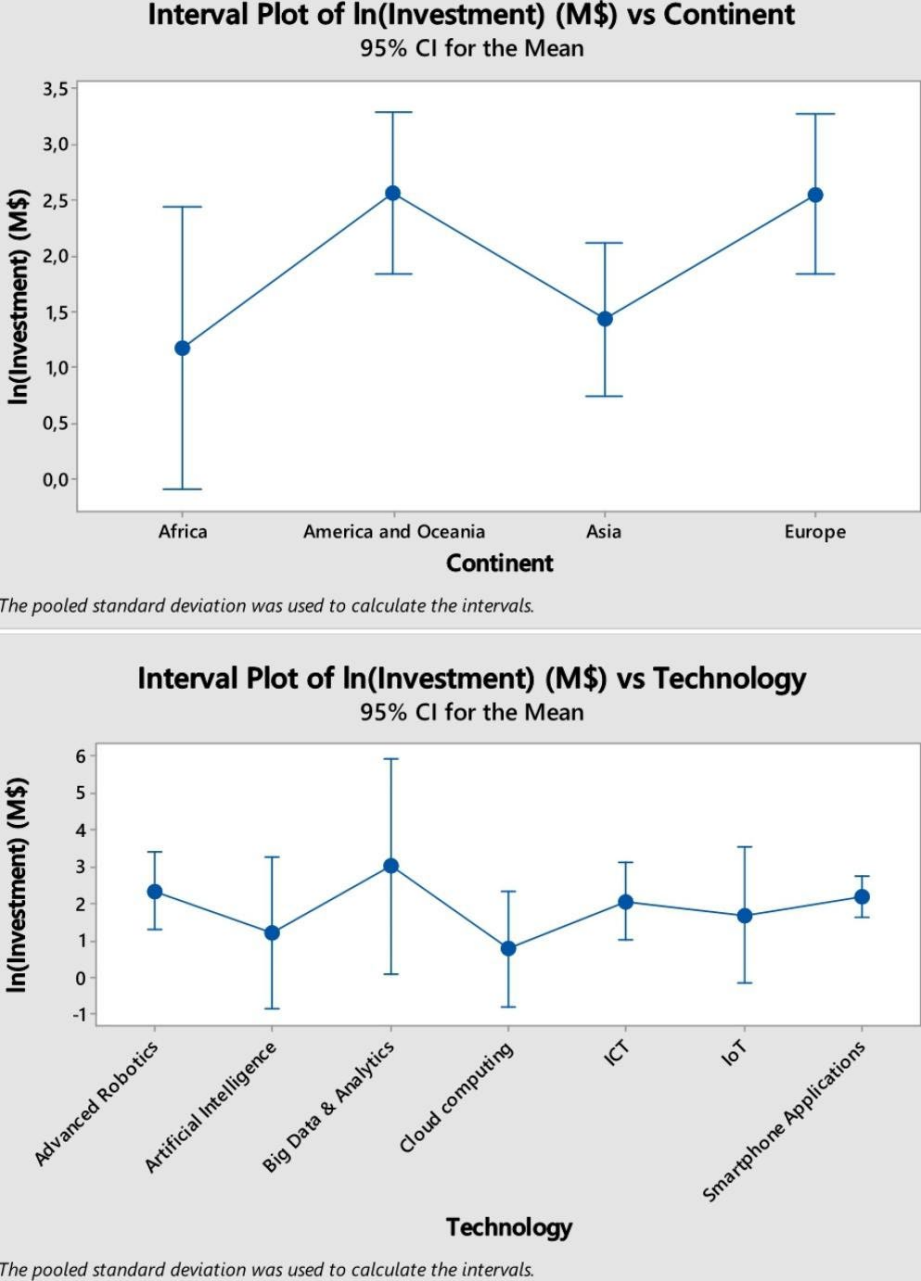
Graphs obtained by conducting the ANOVA

In addition, in order to complement the results of the regression analysis, and thus being able to give a more thorough interpretation of them, an ANOVA considering the technology implemented and the geographical area wherein the projects are developed has been conducted (Figure 5). The resulted p-value associated with the geographical area is equal to 0.033. Thus, there is a significant difference on the average amount of money invested in a project. In particular, America and Oceania, and Europe are willing to invest more money in each innovative project, compared with Africa and Asia even some latecomer countries are closing the gap with the more developed ones in their innovative capacity (Araújo and Salerno 2015). On the contrary, by observing the different technologies considered in the present study, no significant difference is shown. This means that, in terms of amount of money invested in each project, there is not a technology with huger initiatives.

## Discussions and implications

The linear regression analysis carried out focused on the definition of the relationship between the socio-economic configuration of urban areas and the willingness to invest in innovative urban logistics projects. The results obtained after completing the statistical analysis indicate that the implementation of 4.0 solutions in cities context depends on Economic, Social and Urban Structure variables.

The willingness to invest is facilitated in countries with higher levels of income, considered both on a country basis (GDP) and on an individual basis (GDP per capita). Indeed, by observing the sample, it comes up that the countries with high levels of investments in CL 4.0 projects, namely America and Oceania, Europe, show higher values of GDPs. Such a result reflects the expectations concerning the influence of this contextual factor on the willingness to invest in innovation. As a matter of fact, GDP represents the market value of all the final goods and services produced in a certain period of time and it also indicates the level of economic development of a country. Thus, a broad availability of financial resources is also associated to spending for goods that require logistics processes. As a consequence, the investments in more developed areas become more attractive. This is quite relevant. As a matter of fact, GDP and GDP per capita are the only significant factors showing a positive impact on the willingness to invest on innovative CL initiatives. This means, that geographical areas with heavy availability of funding are more willing to invest in logistics urban processes. Such a result might be due to the fact that Industry 4.0 in CL has not been reached a sufficient level of maturity and consequently the related returns on investment are still uncertain (Katsela and Pålsson 2021).

On the contrary, the expenditure in R&D negatively influences the investments in innovative technologies in CL 4.0. This result is not coherent with the expectations. However, the negative relationship might be due to the fact that businesses and governments investing in R&D are keen to apply innovative solutions in other countries, encouraged by lower costs or increased market opportunities (Thukral 2021). As a matter of fact, innovations developed in regions with intense R&D activities, and especially the most disruptive ones, often do not find a favourable environment in their original geographical areas. This because those areas are represented by developed countries with high implementation costs or the local market is not yet ready to welcome so novel innovations. Therefore, the innovation applications in such countries might be very few and sometimes limited to pilot projects without consequent large-scale implementations. Furthermore, underdeveloped countries and urban areas with lower levels of R&D expenditure might represents more promising contexts in terms of development potentialities for private and public promoters interested in investing in innovative CL solutions. Additionally, the negative influence of R&D expenditure may be related to the circumstance that CL 4.0 is a recent phenomenon and Industry 4.0 technologies are more established in other sectors, such as manufacturing. The novelty of the CL 4.0 notion suggests the need for further exploring the potential for applying Industry 4.0 technologies to CL processes by exploiting the knowledge already acquired through their implementation in several manufacturing industries. Of course, effective CL 4.0 interventions will require adapting such a knowledge to the peculiarities and the requirements of the new field of application. In spite of that, the readjustment to different contexts might not imply such a high investment in terms of R&D like the ones needed to develop the technology from greenfield. In this way, the negative relationship of R&D may be justified.

Also, the Employment rate is negatively related to the response variable. This means that urban areas with lower rates of employment are more favourable environments to carry out innovative investments in CL. The present outcome might be due to the fact that governments are trying to support weaker economic territories via different form of incentives such as public subsidies to the investments, and this could make such socio-economic contexts more attractive for promoters (Katsela and Palson 2021). In addition, innovative technologies are often capital-intensive based programs requiring significant investment costs (Hasan et al. 2021). Therefore, it may happen that CL 4.0 solutions replace human resources, with consequent impacts on the employment rate. However, in the next future there will be new jobs resulting from the exploitation of new technologies in the digitalization field (Fareri et al. 2020).

Then, the Urban Population shows a negative impact on investments. The result suggests that lower populated environments are more attractive environments to carry out projects exploiting innovative CL technologies. This is probably due to the fact that less densely populated areas are better able to host and test the deployment of new CL 4.0 technologies. This result is confirmed by real case applications. For example, airborne Drones are currently successfully used in countryside areas. Such an evidence can be also related to the fact that the significant level of complexity associated with high populated contexts make still not effective the delivery operations carried out with robotized solutions (Macrina et al. 2020). Furthermore, small urban areas could be less developed in terms of infrastructures and thus the development of new projects is expected to be more relevant and attractive for private and public investors.

Finally, by considering the geographical area, results reveal that western areas of the world appear to be more engaged in invest in innovative urban logistics initiatives. This might be due to a more mature urbanization that in recent years has been called for solutions for dealing with congestion and pollution problems. However, the dramatic growth of Asian countries in developing innovation, is expected to rapidly bridge this gap.

Based on the results obtained from the empirical analysis, both theoretical and practical implications can be traced. From a theoretical perspective, the present work offers a preliminary overview of the main contextual variables affecting the engagement in CL innovations. As a matter of fact, most of the literature is focused on analysing the operational and the financial feasibility aspects of CL projects. On the contrary, the proposed research might be a first attempt to initiate a stream of literature about the role of external variables in the spread of innovative projects developed in the CL field. This is supported by a quantitative analysis carried out at an international level in countries with different structural and geographic characteristics. In such a way, the present study is also a support to the development of the notion of Logistics 4.0 (Jahn et al. 2018) in specific scenarios, such as the urban ones. In addition, the developed study might benefit the literature on Industry 4.0 technologies by stimulating researches on possible new fields of applications that are not only related to the manufacturing sector where the Fourth Industrial Revolution was initiated. In particular, this contribution can foster investigations about the social and economic conditions that might play a prominent role in facilitating the enlargement of the 4.0 paradigm to those service sectors that are currently more in need for keeping a high level of efficiency by exploiting cutting edge innovations. Finally, the nature of the CL service implies a tight interconnection with human, sustainability, and resilience issues, the three core values of the emerging Industry 5.0 notion. Thus, the present research might drive future studies investigating the application of digital technologies to urban freight transportation so that customer satisfaction is enhanced by for example increasing flexibility and minimizing pollution as well as energy consumption.

From a practical point of view, the proposed analysis might support private promoters in figuring out suitable business areas wherein concentrating their efforts, that is those urban contexts that seem to be more promising for investing in CL 4.0 initiatives based on the results obtained from this research work. At the same time, public agencies might exploit this work, in order to better understand their potential to foster new CL projects and in turn to identify more favourable business environments for carrying out investments in innovative technologies. Thus, this could also help both practitioners and policy makers in more properly design strategies for innovating the existing CL systems. Such an aspect is acquiring a crucial importance, especially during the Covid-19 pandemic. In fact, in some areas of the world, public agencies are financially promoting and supporting innovative projects aimed at improving environmental sustainability in logistics. Its enhancement is expected to have also positive impacts on liveability of a city and more in general the quality of life for the citizenship. In fact, a more developed CL system might influence the mobility, via reducing the traffic and the congestion, the street safety with lower rates of traffic accidents and lower levels of pollution not only in terms of gas emissions but considering both noise and vibrations. In this context, the identification of several socio-economic variables that might impact on the willingness of investing becomes certainly particularly interesting. For instance, the Next Generation Europe Program is promoting the European Green Deal for limiting the global climate change through the support of sustainable initiatives in logistics, that might be adapted to the future digital age (Europa 2021). Moreover, the CL projects reviewed in this study might support providers of 4.0 technologies to have a deeper understanding of the current state of their applications to urban logistics, together with the particular requirements of such a setting. This might be useful for further development and refinement of 4.0 technologies to make them suitable to multiple contexts. Digital technology providers also might benefit from the proposed study in order to identify what application fields and specific technologies are more likely to become popular in future and under what contextual conditions.

## Conclusions

The present work investigates the contextual factors that might drive investments in CL 4.0 initiatives. To this end, a dataset of 105 records based on the primary collection of urban CL projects exploiting the main 4.0 technologies is here presented. The projects included in the dataset refer to innovative solutions developed in the last decade. The data were collected via primary open sources and reports showing the implementation of innovative CL projects and the investment required for developing the project.

In addition, since the aim of this research is analysing the relationship between the implementation of 4.0 logistics solutions in urban areas and the socio-economic factors characterizing the geographical area of development, data related to GDP, GDP growth, Foreign Direct Investments, R&D Expenditure, Employment rate, People with a Bachelor degree, Population Density, Number of Inhabitants, and Internal Private Credit were gathered via other official sources such as Statista and World Bank.

After that, the dataset was analysed in terms of geographical distribution of the projects, level of diffusion of each 4.0 technology and the average investment carried out to develop such innovative solutions. It turns out that the majority of projects are developed in Asia, followed by Europe and America, while the technology most implemented are Smartphone Applications, ICT & ITS, and Advanced Robotics. Finally, the solutions requiring the highest levels of monetary investment are ICT & ITS, Additive Manufacturing and Big Data & Analytics.

Additionally, an empirical regression model was completed in order to identify the main relationships among the level of investments in CL 4.0 programs and several contextual variables. The results show that the implementation of 4.0 solutions in cities context depends on Economic, Social, and Urban Structure variables, since the statistical analysis demonstrates that GDP, GDP per capita, R&D Expenditure, Employment Rate and Urban Population are significant drivers to invest in 4.0 technologies applied to CL. In particular, the level of economic development positively drives the implementation of new CL projects. On the contrary, the attention posed on R&D, the portion of working population, and the size of urban areas in terms of number of inhabitants jeopardize the growth for 4.0 CL initiatives.

Thus, the novelty of the present work coaches on the analysis on the combined effects of different contextual variables that are expected to drive the investments in innovative CL projects. To this end, an empirical approach was selected and applied to a dataset of real projects that were completed in the last years. Therefore, the results of the analysis even referring to a limited sample of projects, might be spread and they can be considered as reliable for future investigations.

Even though the promising results, this study suffers from some limitations. First, the combined effect of the contextual drivers taken into account, together with several operational variables of the private investors was not investigated. For instance, the financial robustness of the promoters was not examined. This aspect could be quite interesting, as it could be a lever for facilitating the implementation of new initiatives. In addition, the level of success of the projects of the sample was not assessed. In fact, the presented research does not consider the main effects of each project with the related managerial and operational impacts on the company’s business, which can be either positive or negative.

Thus, future studies will be addressed in analysing the effects of 4.0 CL projects in urban areas, by including internal business factors. Also, in order to consider the main implications at managerial and operational levels, the success degree of each project, as well as the satisfaction of the involved stakeholders, will be investigated. Finally, future contributions might be built on the obtained results to address the interconnections between the latest industrial innovations, including the emergent Industry 5.0, and the logistics sector. Among them, it might be interesting to compare the outcomes of the present study carried out at an international level with those of vertical national investigations, in order to appreciate any possible differences in terms of the most relevant 4.0 technologies, as well as their key enabling factors according to the CL strategies applied in each country. As part of this research stream, why and how the level of R&D activities influences the willingness to implement CL 4.0 innovations might also be deepened.
